# On the Breathability of Epidermal Polymeric-Printed Tattoo Electrodes

**DOI:** 10.1021/acsaelm.4c01902

**Published:** 2025-02-05

**Authors:** Marina Galliani, Francesco Greco, Esma Ismailova, Laura M. Ferrari

**Affiliations:** † The Biorobotics Institute, Scuola Superiore Sant’Anna, Pontedera 56025, Italy; ‡ Mines Saint-Etienne, Centre Microélectronique de Provence, Gardanne 13120, France; § Department of Excellence in Robotics and AI, Scuola Superiore Sant’Anna, Pisa 56127, Italy; ∥ Interdisciplinary Center on Sustainability and Climate, Scuola Superiore Sant’Anna, Pisa 56127, Italy; ⊥ Institute of Solid State Physics, Graz University of Technology, Graz 8010, Austria; # INRIA, Universite Côte d’Azur, Sophia Antipolis 06903, France

**Keywords:** epidermal electronics, tattoo electrodes, breathability, PEDOT:PSS, wearables

## Abstract

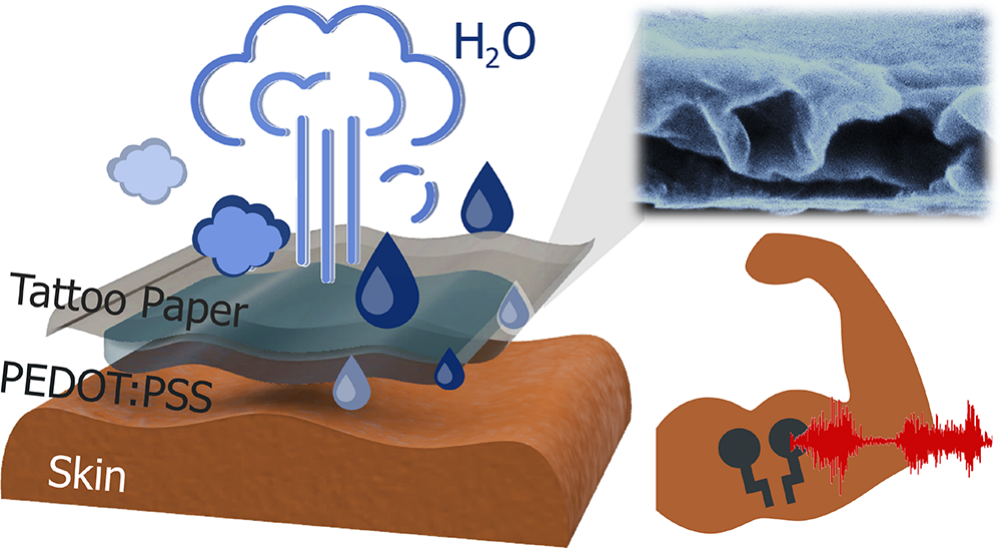

Tattoo sensors offer many of the features of next-generation epidermal devices. They are ultrathin and conformable electrodes that have been shown to record high-quality biosignals from the skin. Moreover, they can be fabricated through large-area processing such as printing. Here, we report on printed poly(3,4-ethylenedioxythiophene) polystyrenesulfonate (PEDOT:PSS) tattoo electrodes breathability. Epidermal devices require a breathable interface to ensure a physiological transepidermal water loss for reduced skin inflammation and discomfort of the user. In this work, we deeply examine the polymeric tattoo sensor’s permeability properties with complementary experiments. By assessing the water permeance, the water-vapor transmission rate, and the impedance spectroscopy of polymeric tattoo electrodes, we show that they are intrinsically breathable, establishing a dry interface with the skin. The stability of such a dry interface is shown through the recording of muscle activity during sport when the sweat rate is much higher. While breathability is often hindered in conventional epidermal sensors, in PEDOT:PSS tattoo electrodes, it lies at the core of a stable sensor performance.

## Introduction

The next generation of wearables demands breathable interfaces to enable long-term and inflammatory-free recordings.^1,2^ A breathable interface significantly reduces stuffiness, rashes, and other inflammatory skin reactions compared to conventional systems supported on plastic and elastomer films, which possess low gas permeability.^2,3^ Breathable skin-worn devices avoid the accumulation of sweat at the interface, permitting the physiological transepidermal water loss (TEWL) from the skin to the surrounding environment.^4^ Differently, an impermeable device causes the entrapment of the skin perspiration; a layer of sweat forms at the interface within a short time. A steady sweat layer results in skin irritation and itching. Moreover, the skin adhesion and the signal stability are compromised due to the possible sliding and even detachment of the device from the skin.^5^ The sweat layer locally changes the skin hydration level, causing an altered data acquisition, for example, hampering the capability of the sensor to measure actual skin impedance variations. In the field of health biomonitoring, wet Ag/AgCl electrodes are the standard reference, while they show serious limitations regarding wearability and long-term stability.^6^ Wet electrodes are uncomfortable; the recorded signal is stable only in the short term, and they can cause skin irritation. Dry and conformable epidermal electrodes have been developed to overcome these issues.^7,8^ The most used substrates for dry sensors are PET, PEN, Parylene C, and silicones.^9,10^ When looking at the breathability properties, these substrates show poor intrinsic gas permeability. The water vapor transmission rate (WVTR) is between 0.4 and 4 g m^–2^ h^–1^.^10,11^ To increase the breathability of such epidermal sensors, perforation of the substrate has been proposed. Indeed, adding porosity increases the permeability of the biosensor.^2,10,12^ In a recent work, a soft silicone adhesive layer is microperforated, reaching a WVTR of around 10 g m^–2^ h^–1^. Here the direct correlation between thickness reduction and the increase of WVTR is reported.^10^ Ag-TPU tattoo-like electrodes showed high breathability reached by using porous TPU obtained through a multistep protocol.^13^ Holey graphene tattoos, made of graphene/PMMA/tattoo paper, have also been fabricated to achieve a WVTR of 115 g m^–2^ h^–1^.^14^ Similarly, Ag-Parylene C tattoo-like sensors have been rendered breathable by an additional microperforation fabrication step using a photoresist and wet etching.^15^ A metallic PET tattoo-like sensor has been fabricated showing the passage of perspiration around serpentine-shaped electrodes.^16^ In all these methods, the fabrication is quite complex, demanding multistep procedures.

As an alternative, intrinsically porous substrates such as paper, fabric, and electrospun nanofibers have been reported as ideal materials to address the challenges of breathability, flexibility, and robustness of next-generation wearables.^17,18^ A built-in water-vapor permeable sensor is first reported by Someya’s group as a nanomesh conductor.^2^ Highly gas-permeable conductive nanomesh structures have been developed for temperature, pressure, and motion monitoring.^17,18^ The high permeability is reached thanks to the internal structure obtained via the electrospinning technique.^19,20^ Recently, a hybrid metal–polymer electrode, made of silver nanowires-PEDOT:PSS (AgNW/PEDOT:PSS), has been reported, showing the intrinsic high water vapor permeability of PEDOT:PSS nanofilms.^21^ The AgNW/PEDOT:PSS electrodes are made through different steps of fabrication, including spin-coating, plasma activation, and lift-off in water. In all of these examples, the main limitation is related to the manufacturing process, which is not easily scalable and needs multiple steps. The breathable properties came at the cost of an increased fabrication complexity of the final device, limiting the scale-up of the technology.

In this paper, we investigate in-depth and prove the intrinsic breathability properties of fully polymeric and printed tattoo electrodes. Tattoo-based sensors exploit commercially available temporary tattoo paper as a substrate. The latter is made of a layered structure of a supporting paper sheet, a water-soluble layer (e.g., starch, dextrin), and a releasable polymeric film (e.g., ethyl cellulose, thickness ∼0.5 to 1 μm). The releasable polymeric film can be functionalized with conductive materials, such as PEDOT:PSS,^22^ graphene,^14^ and carbon.^23^ Polymeric tattoo sensors are ultrathin and dry (gel-free) featuring conformability, self-adhesion, long-term mechanical stability under stretching (up to 96 h^24^), and recording stability when worn on the skin (up to 48 h^25^). All these features are combined with the key advantages of easy handling and fast, large area, and low-cost fabrication.^24,26^ This work proposes a methodological approach to study the breathability of polymeric-printed tattoo electrodes that differs from state-of-the-art studies where only the water-vapor passage is assessed through the evaluation of the WVTR. Here, we show a procedure to study both the passage of vapor and liquid water through multiple experiments. Notably, liquid water has a higher molecular density with respect to that of water vapor, which is a relevant aspect when water permeability is investigated. At first, tattoos are examined through scanning electron microscopy (SEM) and atomic force microscopy (AFM) micrographs, showing the presence of cavities in the internal structure. The profile section of tattoo sensors has already been observed by SEM^27^; however, no internal structure investigation was reported. Then we study the liquid water permeance through a standardized setup for membrane assessment. This confirms the hypothesis of a porous internal structure, as the films show compressible behavior. Then, the WVTR is evaluated. We compared the WVTR to the TEWL, showing that the physiological perspiration is unaffected. Electrochemical impedance spectroscopy (EIS) is reported for wet, PET-based, and polymeric tattoo electrodes. The tattoo interface remains dry over time (2h), differently than other electrodes, showing that tattoos can exchange liquid water and water vapor with the ambient. Finally, electromyography (EMG) is recorded during physical exercise to showcase the stability of the tattoo-skin dry interface also when the perspiration level exceeds the TEWL.

## Results and Discussion

**Figure 1:**
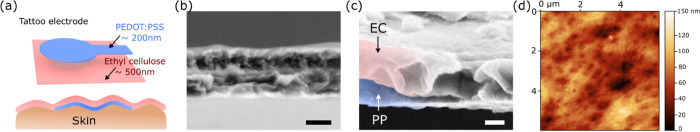
Tattoo electrodes macro- and microstructure. (a) Graphical representations of the tattoo electrode bilayer structure in its exploded view (top) and cross-section view (bottom) when transferred on the skin. (b) SEM micrographs of the cross-section and (c) tilted cross-section (∼10°) of a tattoo electrode (swollen state) highlighting its internal structure and the ethyl cellulose (EC) - PEDOT:PSS (PP) bilayer. Scale bars represent 500 and 200 nm in (b) and (c), respectively. (d) AFM image of an EC tattoo nanofilm showing the surface topography.

Tattoo electrodes are fabricated by printing the organic semiconductor PEDOT:PSS onto the top releasable film of the tattoo paper, resulting in a bilayer of ∼500 nm of ethyl cellulose (EC) and ∼200 nm of PEDOT:PSS (Figure 1a). Once manufactured, at the time of need, the tattoo electrodes are released on the skin by just wetting and removing the backing paper. The laminated tattoo film conforms to the skin texture, and its submicrometer thickness provides for self-adhesive properties. We hypothesized that the skin perspiration produced beneath tattoo electrodes can reach the external environment, passing from the liquid to the vapor phase by percolating the EC pores and passing across the PEDOT:PSS film. To confirm this hypothesis, we inspect the tattoos’ surface morphology and internal structure, we study the water permeability, and we assess the WVTR.

To gain insights into the internal morphology of PEDOT:PSS tattoos, we imaged their profiles with SEM. Figure 1b,c reports the tattoo cross section, where a porous morphology is visible. The tattoo film shows a structure where cavities, of different form factors and shapes, are distributed across the film thickness. The bilayer made of EC and PEDOT:PSS is appreciable in the tilted tattoo electrode profile (Figure 1c). The fine-scale surface morphology is observed by AFM. The AFM measurement of the EC film surface topography (Figure 1d, full-scale range in Figure S1a) shows a rough surface (RMS roughness = 65.4 nm) with depression points with variable shape and dimension. The three-dimensional visualization reported in Figure S1b highlights the presence of cavities, confirming a porous structure of the nanofilm. The irregular morphology and the high variability in pores dimension and distribution found in different EC films (batch-to-batch variability) are revealed by the AFM image of a second EC sample (Figure S1c,d); here, a different roughness (RMS = 42.24 nm) is observed. The grain analysis of the AFM images (Figure S2a–d) suggests a slightly different variability in the pore depth (*Z*_*m*_) distribution of the two studied EC nanofilm samples.

**Figure 2:**
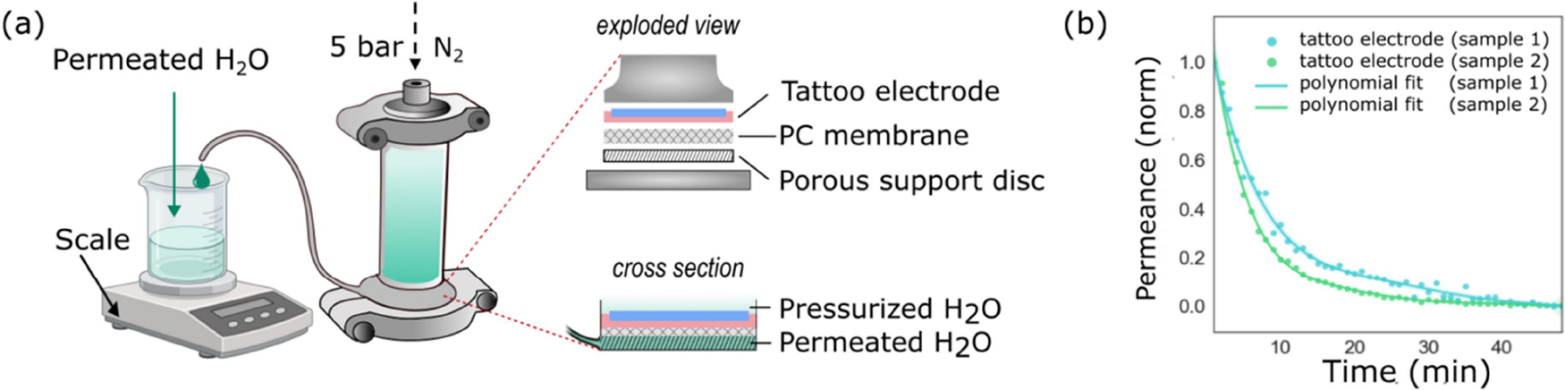
Tattoo permeability to liquid water. (a) Schematic of the experimental setup: the tattoo electrodes (made of EC-PEDOT:PSS bilayer) are supported by a polycarbonate (PC) commercial membrane and mounted on a porous support disc placed at the end of a vertical cell, filled in with a water column, and pressurized. The permeate (water) is collected and weighted on a scale over time to measure the tattoos permeance (left). (b) (Min-max) normalized water permeance of the two tested tattoo electrodes reporting the sampled (points) and the fitted (lines, 6° polynomial) data.

We study the tattoos’ permeability to liquid water with a standardized setup for membrane evaluation (Figure 2a). We assess the water permeance P [L m^–2^ h^–1^ bar^–1^] on two samples (Figure 2b). The results confirm that liquid water can pass from the column into the tattoo voids to reach the surrounding environment. A monotonically decreasing flux of water is observable over time with its maximum as soon as the experiment starts. From the permeance decreasing over time, the compressibility of the sample can be deduced,^28^ which is linked to the cavities collapsing. The permeance decline is a sign of the film compacting due to the progressing collapse of the pores subjected to applied pressure. From the first three data points, where the compressibility level is at its minimum, we extrapolate the water permeance P of 7.6 ± 0.6 (L m^–2^ h^–1^ bar^–1^) for one sample and of 46 ± 6 (L m^–2^ h^–1^ bar^–1^) for the second sample. The difference in the P is due to the natural random pore distribution in both the EC and PEDOT:PSS layers. Nevertheless, by normalizing the permeance (min-max normalization) and fitting the acquired data with a polynomial curve (6°, 95% confidence interval), an equal trend in the tested electrodes is visible (Figure 2b). This result confirms that the tattoo electrode allows for liquid water passage. To visually show the water transfer through the tattoo interface, we laminated a tattoo electrode onto a syringe and manually applied some pressure. This customized setup empirically exhibits the water passage through the film (Supporting Information, Figure S3).

**Figure 3:**
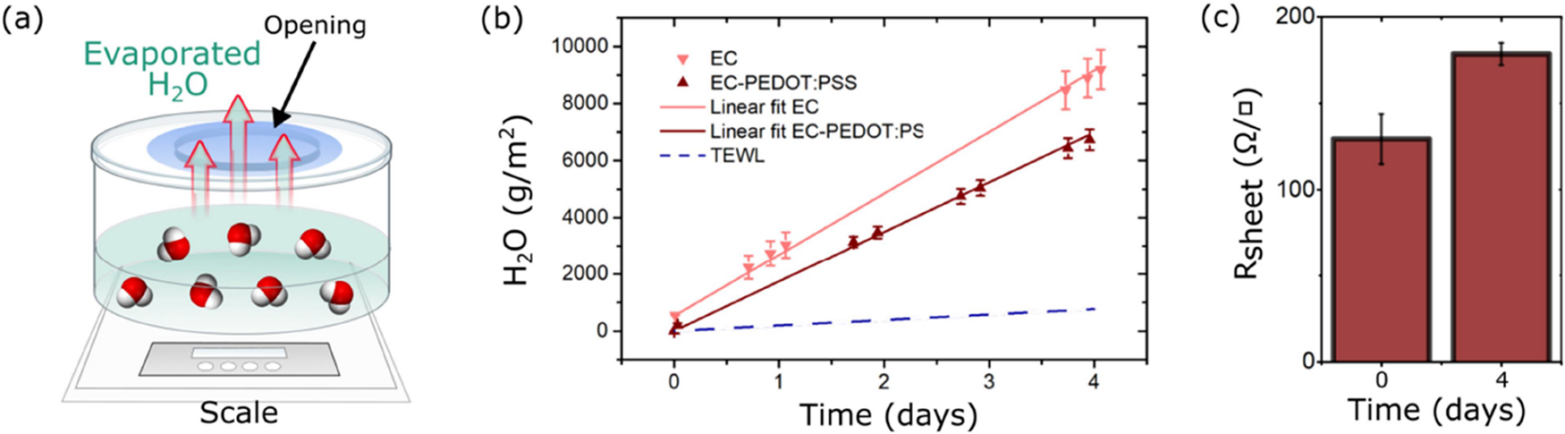
Tattoo permeability to water vapor. (a) Schematic of the WVTR measurement setup: the tattoo electrode is laminated on top of the opening of a plastic jar filled with water, and the evaporated water loss is measured through a scale. (b) Results of the measured water loss over time. The WVTR is extrapolated from the linear data fitting. (c) Measurement of tattoo electrode sheet resistance at days 0 and day 4.

Then, we study the WVTR. Using a dedicated experimental setup, the water-vapor passage through the tattoo is monitored over time (Figure 3a). In this experiment, we compare the pristine EC film and the tattoo electrodes made of EC-PEDOT:PSS. The WVTR of the pristine EC film is found to be 85 g m^–2^ h^–1^, while for the EC-PEDOT:PSS tattoo electrodes, it is reduced to 70 g m^–2^ h^–1^. The WVTR is extrapolated from the slope of the linear regression (*R*^*2*^ = 0.99) of the water loss per unit area and time, as presented in Figure 3b. This is consistent with the results obtained in a similar experiment, with a different tattoo paper type (∼96 g m^–2^ h^–1^).^21^ With respect to the state of the art, the WVTR of tattoo electrodes lies in between the WVTR of microperforated silicone-based electrodes, which equals 11 g/m^–2^ h^–1^,^10^ and of holey graphene/PMMA tattoos which is 115 g m^–2^ h^–1^.^14^ Looking at the difference between EC and EC-PEDOT:PSS films, we observe a decrease in WVTR of around 17%. This can be related to two main factors. First, the deposition of the PEDOT:PSS layer increases the tattoo thickness and the total film resistance to vapor passage. The WVTR decreases due to the inverse relationship between the film thickness and permeability. Second, it is known that the PEDOT:PSS can swell, thanks to the deprotonation of the sulfonate groups in PSS-rich regions.^29,30^ The evaporated water, without ions, is absorbed by PEDOT:PSS. This is confirmed by the tattoo electrode sheet resistance increase of +38% between day 0 and day 4 (Figure 3c). The water molecules swelling the PEDOT:PSS film cause an increase of the physical distancing between PEDOT conductive grains and thus a rearrangement of the conducting paths, in turn resulting in the conductivity decrease.^31^ Water swelling of PEDOT:PSS films has been deeply investigated. PEDOT:PSS films, when immersed in water, can increase their mass by 40–600%, depending on the PEDOT:PSS blend composition and experimental setups.^32,33^ The water uptake of EC-PEDOT:PSS tattoo electrodes has been estimated by comparing their thickness in a dry (608 ± 52 nm^11^) and swollen status (885 ± 22 nm). The thickness of the swollen status was measured from the SEM image of Figure 1b. An increase of 42% is found. The increase in the thickness of swollen samples is proportional to the increased samples’ resistivity, as reported in Figure 3c. Finally, by comparing the tattoo WVTR (∼70 g m^–2^ h^–1^) with the normal TEWL (4–8 g m^–2^ h^–1^^34^ for a healthy adult), we found it to be 1 order of magnitude higher. This outcome demonstrates that tattoo electrodes are breathable, letting the skin perspire without altering physiological perspiration.

**Figure 4:**
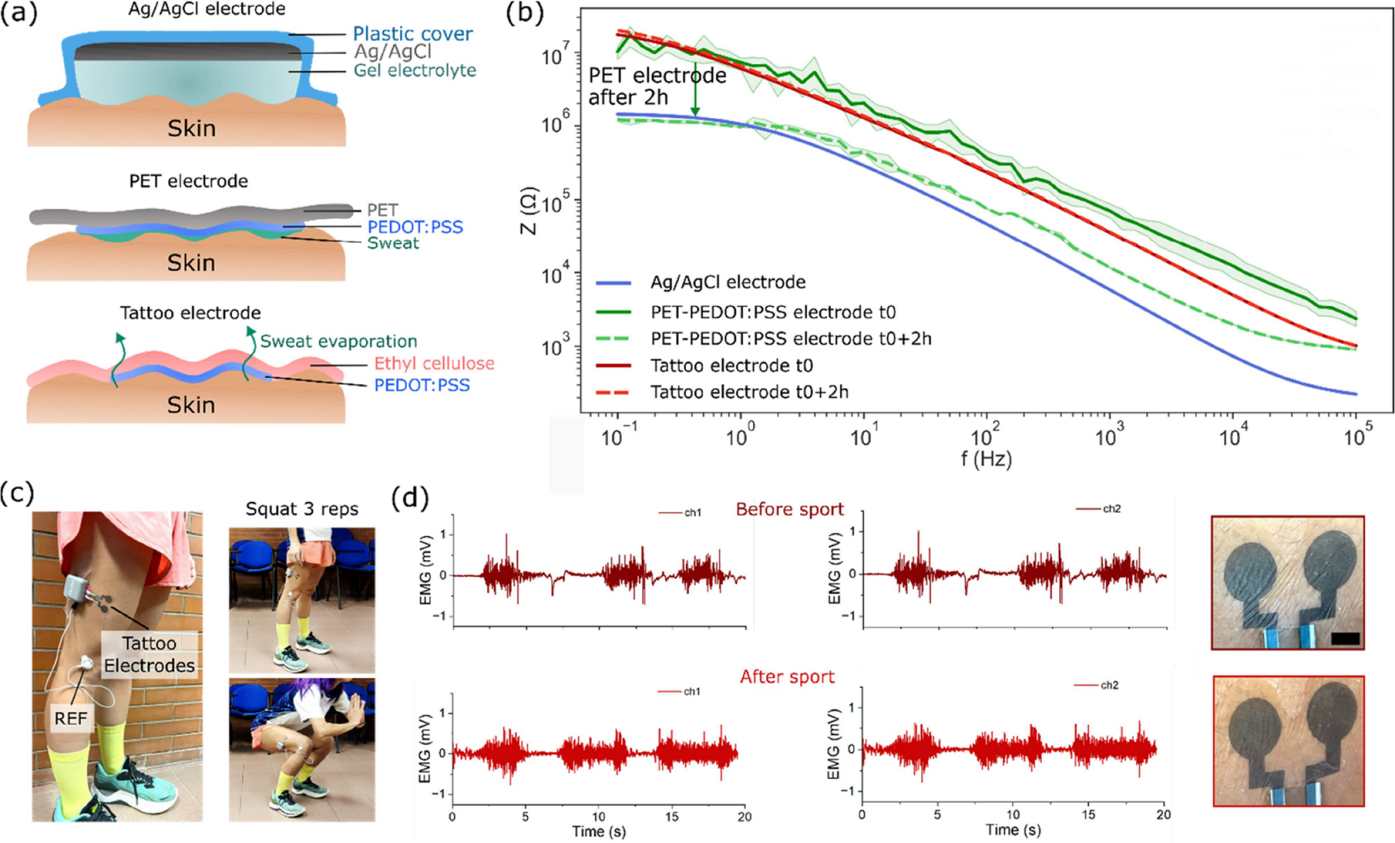
Electrode-skin interface. (a) Schematic of the Ag/AgCl, PET, and tattoo interfaces with the skin characterized by the presence of a gel electrolyte, an accumulated sweat layer, and a breathable dry interface, respectively. (b) Impedance magnitude of the tree electrodes on the skin at *t*_0_ and *t*_0+2h_. The shaded area under the lines represents the standard deviation of the data. (c) Tattoo electrodes on the vastus lateralis muscle. (d) Left: the EMG recordings from the tattoo electrodes before and after 30′ of sports activity. Right: Photographs before and after the sports activity of the PEDOT:PSS tattoo couple were used for the recording. The scale bar is 5 mm.

EIS of electrodes worn on the skin is carried out at two different times (t_0_ and t_0+2h_) to show that breathability is all along ensured. During this time frame, perspiration is produced and would eventually be entrapped under the electrode in the case of poor breathability. EIS of PEDOT:PSS tattoos, PET–PEDOT:PSS electrodes, and standard wet Ag/AgCl electrodes are compared (Figure 4a). The acquired impedance moduli are reported in Figure 4b. The Ag/AgCl electrode impedance shows the typical plateau at low frequencies, proper of the RC circuit that models the wet sensors.^35^ At t_0_ the PEDOT:PSS- tattoo and PET electrodes do not show this plateau, which is evidence of a more capacitive response of the dry electrodes. However, just after a few minutes, perspiration is produced, and a thin sweat layer accumulates at the PET electrode-skin interface. As previously reported, dry electrodes are designed to operate without an explicit electrolyte. Instead, it is usually supplied by moisture on the skin (i.e., sweat). The impedance of dry electrodes can be quite comparable to wet electrodes after a few minutes due to sweat and moisture buildup.^36,37^ The PET substrate is indeed quite impermeable to either liquid or vapor water.^38^ This occurrence is visible from the shift of the impedance modulus from the capacitive dry-electrode trend (solid green line) to the wet-electrode trend (dotted green line) at t_0+2h_ with the characteristic plateau at lower frequencies. Here, the accumulated sweat operates as a gel in the Ag/AgCl electrodes, making the impedance of the two devices comparable at all. Conversely, the tattoo electrode spectrum (red) does not differ from t_0_ to t_0+2h_, showing a constant and stable impedimetric response over time. Tattoos exhibit higher signal stability, which is instrumental in reliable long-term applications. As previously reported, compared to wet electrodes, tattoos transduce physiological signals through a capacitive coupling with the first layers of the skin,^25^ allowing the acquisition of challenging signals (e.g., electroencephalography^22^) despite having a relatively high impedance modulus.

Finally, to showcase the breathability of tattoo electrodes, we record and compare the signal quality of EMG before and after sport. During physical exercise, the sweat rate is much higher than the normal TEWL (between 697 and 1710 g m^–2^ h^–1^^39^), thus the need for a breathable interface is crucial. A tattoo electrode with two channels has been placed on the vastus lateralis muscle and connected to a wearable acquisition unit (Figure 4c). The subject performs 30′ of bodyweight training, and the EMG of a three-repetition squat is acquired and compared before and after the sport (Figure 4d). The mean SNR from the two channels is equal to 36.90 before and 22.68 after sport. The signal quality is comparable, and the tattoo interface remains visibly dry, as is appreciable in Figure 4d (right). The ability of tattoos to acquire EMG signals also during physical exercise is reported in Supplementary Video 1.

## Conclusions

We comprehensively study the permeability of polymeric-printed PEDOT:PSS tattoo sensors to quantify their breathability feature. Tattoo electrodes are intrinsically breathable due to their material composition, low thickness, and film morphology, featuring a porous structure. The permeability of tattoos to liquid and vapor water is quantitatively assessed by evaluating their water permeance *P* and WVTR. Both liquid water (*P* = 26.8 g m^–2^ h^–1^ bar^–1^) and water vapor (WVTR = 70 g m^–2^ h^–1^) can pass through the polymeric printed tattoo electrodes. These values are well above the average human perspiration rate quantified as TEWL = 4–8 g m^–2^ h^–1^.^34^ We ascribe the high water permeability of the polymeric tattoos to two main factors: (i) the porous structure of the polymeric tattoos, observed through SEM images and deduced from the film compressibility, which promotes the water percolation and evaporation at the tattoo interface with the ambient, and (ii) the PEDOT:PSS water absorption due to the PSS hygroscopicity, which at the interface with the air evaporates. The tattoo paper is porous; therefore, it intrinsically has passages/vias the water can enter, pass through, and reach the interface with the ambient. Moreover, the absorption of the perspiration is possible thanks to spontaneous PEDOT:PSS swelling due to PSS hygroscopicity. In this way, the tattoo-ambient interface corresponds to the liquid-water and air interface. Here, because the partial pressure is lower than the vapor pressure, water molecules spontaneously leave the tattoo film and evaporate into the vapor phase. At the skin-tattoo interface, the transepidermal water is continuously absorbed because of the PEDOT:PSS swelling since at the tattoo-ambient interface the water transition from liquid to vapor phase is dynamically promoted. Not only do tattoo electrodes remain dry over time when worn on the skin, as evidenced by EIS experiments, but they also remain stably dry during physical exercise when the sweat rate is much higher. This feature differs from those of other flexible dry electrodes, where the development of a wet interface is visible within a few minutes. The complete study here reported highlights the intrinsic breathability of PEDOT:PSS tattoos, suggesting their use as skin-tolerant epidermal sensors for prolonged body monitoring even during sport.

## Materials and Methods

### PEDOT:PSS Tattoo Electrodes

The electrodes are fabricated as previously reported.^40^ Briefly, they are made through inkjet printing (Dimatix DMP-2800 system, Fujifilm Corp., Japan) of PEDOT:PSS ink (Clevios PJet 700 by Heraeus) on tattoo paper (Tattoo 2.1, by The Magic Touch Ltd., UK). Inkjet printing is carried out with a 10 pL cartridge (DMC-11610), and the PEDOT:PSS ink is used after filtration (Minisart, average pore size 0.20 μm, Sartorius). After printing, the patterned tattoo is dried in the oven (Thermo Scientific OMH 180-S Series) for 15 min at 110 °C. In the case of electrodes for impedance spectroscopy, the sensing area is 1 cm^2^.

### Screen-Printed PEDOT:PSS Electrodes on PET Substrate

The electrodes are fabricated at Printed Electronics Arena, RISE (Norrköping, Sweden) on 50 μm thick PET substrates, purchased from Policrom Screen. The printing of PEDOT:PSS paste (CleviosTM SV4 by Heraeus) is carried out by a DEK Horizon 03iX screen printer under controlled ambient conditions (19–22 °C and 45–55 RH%). The screens (polyester-based) are purchased from Marabu Scandinavia AB. All the screen-printed layers are subsequently dried using a convection oven for 15 min at 110 °C. The sensing area is 1 cm^2^.

### Tattoo Morphology Imaging. SEM Images

To image the tattoo electrodes' internal structure, the sample is submerged in water for 30 min to allow water to enter the cavities (swollen state). Then, the electrode is recollected and dipped in liquid nitrogen. Once frozen, the sample is broken into two parts to get a clear-cut profile and immediately dried in a cooled vacuum chamber. These sample preparation steps prevent the pores from collapsing, allowing the film porosity preservation. The tattoo is then observed through a scanning electron microscope (MEB Ultra 55 Carl Zeiss) after the ∼20 Å carbon layer is deposited on it by sputtering. The SEM built-in software is used to measure the film thickness.

### AFM Images

To perform AFM measurements, EC tattoo films are released from carrier paper and floated in a water bath; they are then recollected and dried on a clean Si wafer. AFM imaging was carried out in the air, at room temperature, using a Veeco Innova scanning probe microscope operating in tapping mode. The system was equipped with Si AFM probes (NSG01, NT-MDT, resonant frequency of ≈150 kHz). AFM images are analyzed to evidence surface topography and roughness with the software GwyddionSPM (available at http://gwyddion.net).

### Water Permeability Test

Permeation tests are performed in a high-pressure filtration device HP4750 (Sterlitech Corporation, USA) with a dead-end configuration using pressurized nitrogen inert gas at 5 bar. The tattoo samples are cut and placed onto a porous substrate and a membrane with negligible flow resistance (0.8 μm polycarbonate porous membrane by Whatman). The tested area is limited with an O-ring of 6 mm diameter (defining an area of 0.28 cm^2^). Then the cell is filled with a column of Milli-Q water (300 mL), and an equilibration time of 10 min is left before applying pressure to the column. This equilibration time is necessary to uniformly hydrate the PEDOT:PSS film under static/passive conditions. The mass of the water permeates is recorded over time with a scale (Fisher-brand) connected to automatic data acquisition software (SPDC data collection). The permeance is calculated by dividing the water flux (Q in L/h) by the active area of the membrane (A in m^2^) and by the pressure ΔP (P in bar) applied during filtration. The data of two samples of tattoo electrodes are reported.

### Water-Vapor Permeability Test

Each sample, EC and EC-PEDOT:PSS, is cut in a circular shape of 7 cm^2^ and released onto a plastic lid, which is mounted on a jar with 2 g of water inside. The jar plastic lid has an opening that defines the working area for the experiments equal to 2 cm^2^ (Figure S4). In the case of PEDOT:PSS tattoos, the film is released so the PEDOT:PSS side is facing the water. The jars are kept at a constant temperature of 21 °C and relative humidity of 40%, and during the whole experiment (4 days), they are weighed daily. The WVTR is extrapolated from the regression curve (OriginLab) of the collected data. The tattoo electrode sheet resistance is measured through a four-point probe setup connected to a source measure unit (Keithley).

### EIS Measurements

EIS (Autolab potentiostat, Metrohm Autolab B.V.) is used to characterize the impedance. The measurements are done in a three-electrode configuration, where the Working (WE) and the Sensing (S) electrodes are connected. In this configuration, the measured impedance consists of two contributions, the impedance of the skin and the skin-electrode contact impedance, related to the area underneath the WE-S electrode. The three electrodes are placed on the skin with a 2.5 cm interspaced center-to-center distance. The counter (CE) and the WE electrodes are Ag/AgCl electrodes (Ambu BlueSensor, REF M-00-S/50) for all the experiments, while the S and the WE are short-circuited and connected to the electrode of interest, tattoo, PET–PEDOT:PSS or Ag/AgCl. In the case of PET–PEDOT:PSS the electrodes are held in position with an elastic band. All the impedance recordings are performed on the forearm of a volunteer, and for each kind of electrode, the measurements are repeated three times, one after the other, except for the recording after 2h. All the electrodes have an area of 1 cm^2^. The measurement is done in potentiometric mode with an applied sinusoidal signal with a 1 mV amplitude. The frequency range was set between 10^–1^ and 10^5^ Hz.

### EMG Recordings

A printed PEDOT:PSS tattoo electrode with two channels is placed onto the vastus lateralis muscle, while a commercial Ag/AgCl medical electrode is placed onto a knee bone to serve as a reference electrode. The electrodes are connected to a wearable acquisition unit (MUOVI, OT Bioelettronica). The EMG signal is recorded from the two channels in a unipolar setup while the subject is performing sport activity. The subject performed 30′ of bodyweight training while wearing the electrodes all along, and the same squat task was repeated to record the EMG before and after the physical activity. The raw signals are filtered with a 50 Hz notch filter and a 1 kHz low pass filter.

### Experiments Involving Human Subjects

This research does not include the collection of identifiable private information related to the individual’s health status and concerns only the technological demonstration. Two able-bodied subjects (females avg age 31 years old) free of any motor disorders participated in this study. Informed consent in accordance with the Declaration of Helsinki was obtained before the experiments were conducted from each subject. The two participants performed impedance recording with tattoo, PET, and Ag/AgCl electrodes.

## Supporting Information Available

AFM images of EC films surface; grain analysis of AFM images; and water passage through EC tattoo nanofilm (PDF)
EMG recordings with tattoo electrodes (MP4)
