# Supplementary material for: On the Breathability of Epidermal Polymeric-Printed Tattoo Electrodes
Source: ACS Appl Electron Mater. 2025 Feb 5;7(4):1408–14. doi: 10.1021/acsaelm.4c01902 (PMC11867139; doi:10.1021/acsaelm.4c01902)
Supplement: Supplementary file 1 — el4c01902_si_001.pdf [file el4c01902_si_001.pdf]

# Supporting Information

## On the Breathability of Epidermal Polymeric-Printed Tattoo Electrodes

Marina Galliani[a,b], Francesco Greco[a,c,d,e], Esma Ismailova[b] and Laura M. Ferrari*[a,c,f]

[a] *The Biorobotics Institute, Scuola Superiore Sant'Anna, Pontedera, 56025, Italy*

[b] *Mines Saint-Etienne, Centre Microélectronique de Provence, Gardanne, 13120, France*

[c] *Dept. of Excellence in Robotics & AI, Scuola Superiore Sant'Anna, Pisa, 56127, Italy*

[d] *Interdisciplinary Center on Sustainability and Climate, Scuola Superiore Sant'Anna, Pisa, 56127, Italy*

[e] *Institute of Solid State Physics, Graz University of Technology, Graz, 8010, Austria*

[f] *INRIA, Universite Côte d'Azur, Sophia Antipolis, 06903, France*

**Corresponding author: laura.mferrari@santannapisa.it*

**Figure S1: AFM images of ethyl cellulose (EC) films surface**

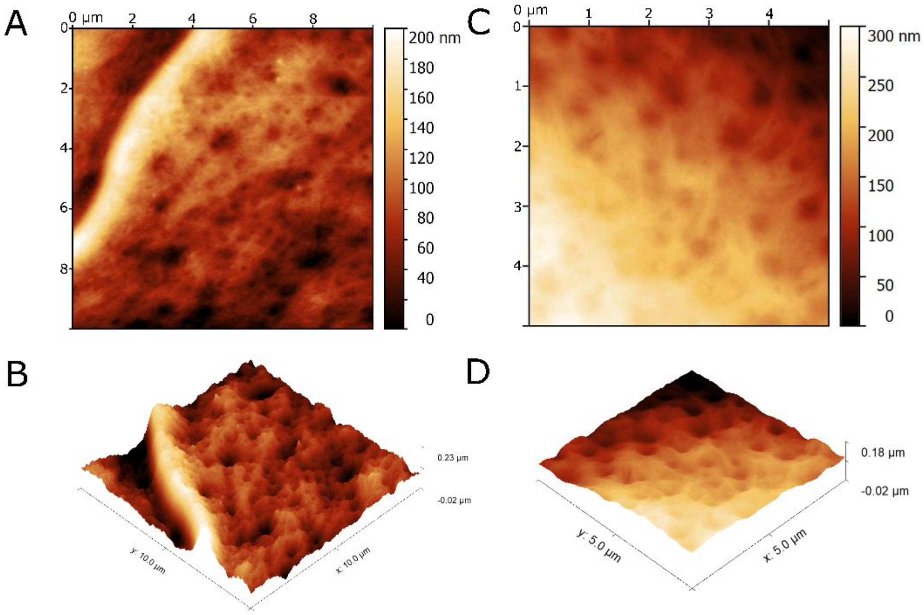


**Figure S1:** A) Full scale range and B) three-dimensional reconstruction of the EC sample topography of Fig. 1D (main text). The high elongated element/stripe, visible at the left top corner, represents a film crease due to the nanofilm folding formed during the sample preparation. C) AFM topography image and D) three-dimensional reconstruction of another EC nanofilm sample from a different tattoo paper kit batch.

**Figure S2: Grain analysis of AFM images**

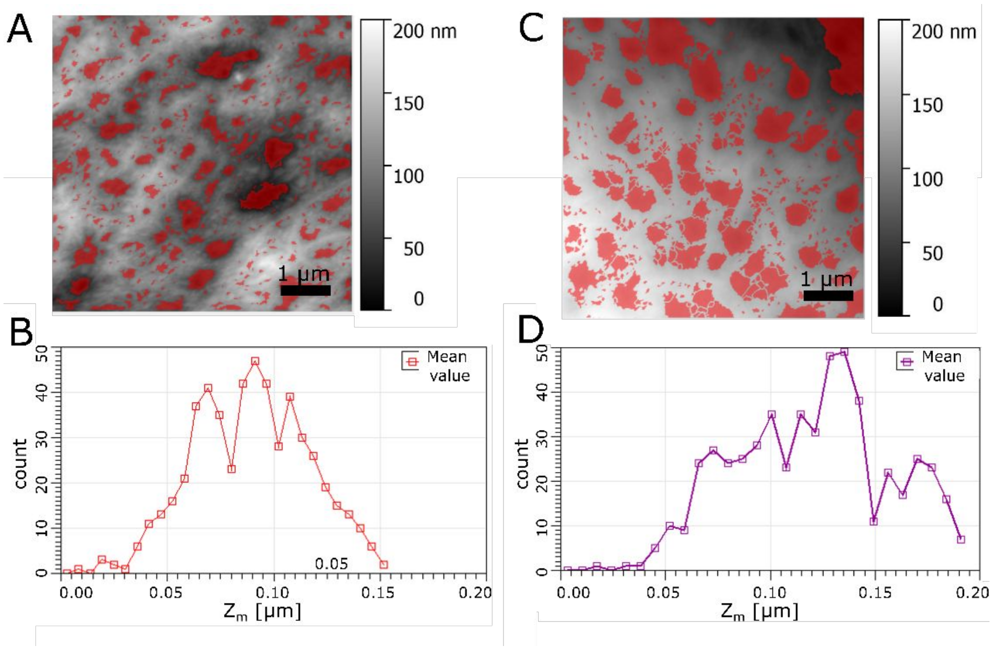

**Figure S2**: A) and B) Grains detection and marking computed with watershed method (Gwyddion software) of the two studied EC nanofilms. C) and D) Distribution of grains with different depths (Zm) calculated by the grain analysis. The grain analysis was performed with the following input values: number of steps = 50, drop size = 1 %, threshold 5 px2 for the grain location and number of steps = 20, drop size = 10 % for segmentation.

**Figure S3: Water passage through EC tattoo nanofilm.**

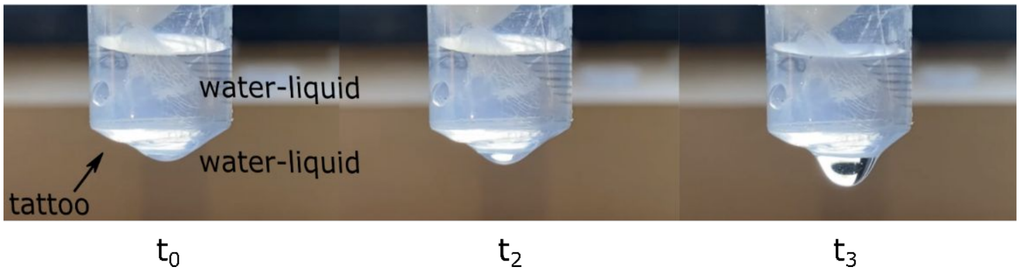


**Figure S3:** Time frames photographs (t0, t2, t3) taken while manually applying pressure on a syringe filled with water. The tip of the plastic syringe was cut out to leave a round opening (diameter c.a. 5 mm). An EC tattoo nanofilm (5x5 cm) was released in a water bath and recollected laminating it on the syringe opening. The borders of the film were tightly glued to the syringe surface to guarantee the water passage only through the EC film on the opening.

**Figure S4: Water-vapor permeability test**

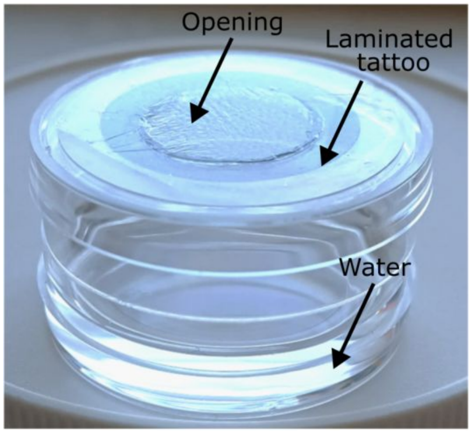


**Figure S4:** Photographs of one of the jars, used for the WVTR experiments, showing an upper opening on which the tattoo is laminated.
